# Structural Insights into the Iodothyronine Deiodinase 2 Catalytic Core and Deiodinase Catalysis and Dimerization

**DOI:** 10.3390/biom14111373

**Published:** 2024-10-28

**Authors:** Holly Towell, Doreen Braun, Alexander Brol, Andrea di Fonzo, Eddy Rijntjes, Josef Köhrle, Ulrich Schweizer, Clemens Steegborn

**Affiliations:** 1 Department of Biochemistry, University of Bayreuth, Universitätsstr. 30, 95447 Bayreuth, Germany; 2 Institut für Biochemie und Molekularbiologie, Universitätsklinikum Bonn, Rheinische Friedrich-Wilhelms-Universität Bonn, 53115 Bonn, Germanyuschweiz@uni-bonn.de (U.S.); 3 Charité-Universitätsmedizin Berlin, Corporate Member of Freie Universität Berlin and Humboldt-Universität zu Berlin, and Berlin Institute of Health, Institut für Experimentelle Endokrinologie, 10115 Berlin, Germany

**Keywords:** crystal structure, type II iodothyronine deiodinase, substrate binding, activity, cross-linking, dimerization interface

## Abstract

Iodothyronine deiodinases (Dio) are selenocysteine-containing membrane enzymes that activate and inactivate the thyroid hormones (TH) through reductive iodide eliminations. The three deiodinase isoforms are homodimers sharing highly conserved amino acid sequences, but they differ in their regioselectivities for the deiodination reaction and regulatory features. We have now solved a crystal structure of the mouse deiodinase 2 (Dio2) catalytic domain. It reveals a high overall similarity to the deiodinase 3 structure, supporting the proposed common mechanism, but also Dio2-specific features, likely mediating its unique properties. Activity studies with an artificially enforced Dio dimer further confirm that dimerization is required for activity and requires both the catalytic core and the enzyme’s N-terminus. Cross-linking studies reveal the catalytic core’s dimerization interface, providing insights into the architecture of the complete, active Dio homodimer.

## 1. Introduction

**Figure 1:**
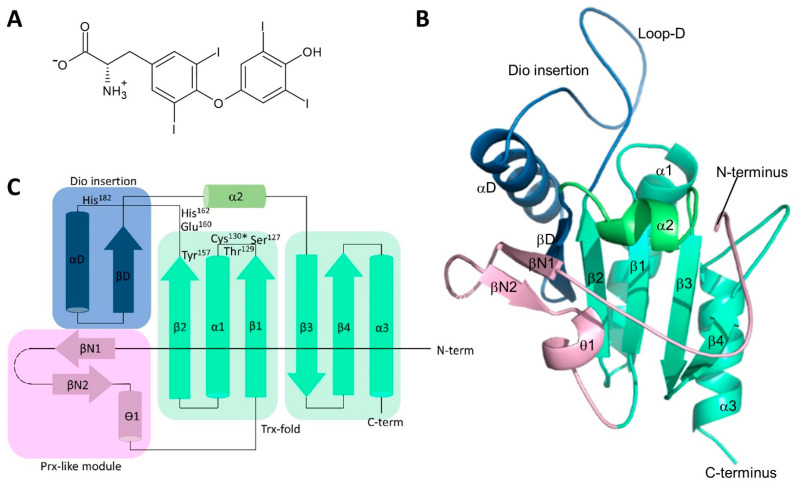
Structure of Thyronines and Dio2. (**A**) Chemical structure of the prohormone 3,3′,5,5′-tetraiodothyronine (T_4_), which can be converted to a variety of thyronines. (**B**) Crystal structure of mouse Dio2 catalytic domain. Secondary structure elements are labeled and colored as in panel (**C**). (**C**) Topology plot for the crystal structure of the mouse Dio2 catalytic domain shown in panel (**B**) The asterisk indicates the catalytic Sec that was mutated to Cys in our construct. The two modules forming the basic Trx fold together with α2 are shown in green. The Trx β-sheet is extended by the strand of the Dio insertion module (blue), which connects it to the β-strands of the Prx-like module (magenta).

The three mammalian iodothyronine deiodinases (Dio1–3) are integral membrane proteins responsible for the local activation and inactivation of the thyroid hormones (TH) [1,2]. The thyroid gland produces predominantly the prohormone 3,3′,5,5′-tetraiodothyronine (T_4_; Figure 1A), which is distributed through the bloodstream to peripheral tissues where it can be 5′-deiodinated into its receptor-activating form, 3,3′,5-triiodothyronine (T_3_). Another Dio-dependent deiodination, at position 5, mediates inactivation of T_3_ and T_4_ and thus limits the effect of TH, e.g., in stem cells. Dios thereby allow cells to fine-tune intracellular levels of active THs to specific needs and contribute to the regulation of tissue development, differentiation, growth, and metabolism [3,4].

Dios feature homologous transmembrane regions and catalytic domains with a catalytic selenocysteine (Sec) residue essential for their unique reductive deiodination mechanism [1,2,5]. A crystal structure of the mouse Dio3 catalytic core (mDio3^cat^) confirmed the predicted thioredoxin (Trx) fold and identified several Dio-specific features and modifications, including a peroxiredoxin (Prx) motif and an N-terminal module similarly seen in 2-Cys peroxiredoxins [6,7]. The active site pocket is formed by a β1-α1-β2 motif of the Trx-fold [6]. A Dio-specific insertion highly conserved among isoforms and essential for substrate binding and/or turnover forms an Ω-loop that borders the catalytic center and contributes to thyronine recognition and a proton relay [6,7], a concept recently further corroborated experimentally in Dio1 [8]. Based on the structure, a conformational change of Dio3-Phe^258^ was proposed, opening a thyronine accommodating cleft next to the catalytic Sec^170^ [6], and molecular dynamics simulations indicate that different conformational states of loop-D enable such a rearrangement [9]. The three Dio isoforms seem to employ a shared deiodination half-reaction mechanism, where the active site selenolate captures the iodine formally as a cation (I^+^), while the thereby reduced substrate uses the electrons of the broken bond for binding a proton [5,8]. The Dio isoforms differ, however, in their regioselectivities: Dio1 can catalyze deiodination of both the inner (tyrosyl) and the outer (phenolic) ring, while Dio2 targets only the outer ring and Dio3 only the inner ring (Figure 1A). The molecular basis of this difference, despite high homology among isoforms, is not yet understood [5,10]. The Dio isoforms seem to share again the general scheme for re-reduction, but also with some less well-understood isoform-specific details [5]. The selenenyliodide either hydrolyzes to an iodide anion and an oxidized selenenyl residue or enters the re-reduction half-cycle directly. The reduction appears to be mediated via a Prx-like intramolecular cascade of selenenylsulfide/disulfide formation that reforms the reduced selenolate and an intramolecular disulfide, which is reduced by an external thiol-cofactor [11,12]. While the artificial cofactor DTT is commonly used in Dio activity studies, experimental evidence implicates glutathione and Trx as the physiological Dio cofactors [6,13,14,15]. Interestingly, Dio2 differs in this context from Dio1 and Dio3 since it actually might not need any re-reduction, or at least is not reduced efficiently. Dio2 is specifically ubiquitinated and thereby inactivated as well as degraded after exposure to its T_4_ substrate, and thus, it appears to perform only a single or very few turnovers [16]. Dio isoforms also differ in their sensitivity to small molecule inhibitors. While all Dio isoforms are potently inhibited by iopanoic acid [17,18,19], gold thioglucose (GTG) [20,21], and xanthohumol [22], only Dio1 is sensitive to inhibition by propylthiouracil (PTU), methylthiouracil (MTU), and genistein [22,23,24,25]. In particular, a Pro residue in Dio2 and Dio3, located two amino acids downstream from the catalytic Sec, seems to protect these isoforms from modification by PTU, but mechanistic details remain unclear [26]. A common feature of Dio enzymes, in contrast, is their homodimeric architecture and that this dimerization appears necessary for catalytic activity [27,28], although controversial data have been published on monomer (in)activity [27,29]. Dimerization appears to be mediated by the transmembrane region and the catalytic domains, but architectural details of dimerization and how it might influence catalysis remain to be clarified [27,29,30].

Dio enzymes feature a generic architecture with three homologous regions—trans-membrane (tm) helix, extended linker, and catalytic core—but also smaller, isoform-specific variations that lead to unique catalytic and regulatory properties for each isoform. Here, we present a crystal structure of the mouse Dio2 catalytic core. Comparison to the previously published mouse Dio3 core identifies Dio2-specific features and provides a structural basis for understanding the generic catalytic mechanisms and the Dio2-specific deviations in regulation and cofactor interaction. Furthermore, we provide experimental results on the relevance and mode of deiodinase dimerization and exploit them, together with the crystal structures, for structural and functional insights into Dio dimerization. Our results thus improve our understanding of Dio architecture and catalysis and will support further functional studies as well as the development of specific, Dio-targeting drugs.

## 2. Materials and Method

All chemicals were from Sigma (Taufkirchen, Germany) if not stated otherwise.

### 2.1. Recombinant Expression and Purification of Catalytic Domain Constructs of Mouse Dio2 and Dio3

The active site Sec was mutated to cysteine in all *Mus musculus* Dio2 and Dio3 constructs. The truncated Dio2 (residues 71–262, or N-terminus as indicated in the text for other constructs) was cloned into pET151 TOPO (Thermofisher, Dreieich, Germany), producing an N-terminal His-tag with a tobacco etch virus (TEV) protease cleavage site. For Dio2, site-directed mutagenesis was performed on two lysine residues (Lys^180^Lys^181^), mutating them to alanine. The truncated Dio3 (78–304) was cloned behind the leucine zipper (LZ) sequence of *A. thaliana* Hy5 (residues 112–150) into pET19b SUMO, producing an N-terminal His-tag with a small ubiquitin-related modifier (SUMO) protease cleavage site, followed by the LZ-Dio3 fusion. The Dio2 protein was expressed in *E. coli* Tuner (DE3) cells (Merck, Darmstadt, Germany) with 1 mM MgSO_4_ for 2–3 h at 30 °C after induction with 0.5 mM IPTG. The LZ-Dio3 protein was expressed in *E. coli* BL21 Codonplus (DE3) (Agilent, Waldbronn, Germany) with 1 mM MgSO_4_ overnight at 20 °C after induction with 0.5 mM IPTG. All cells were disrupted using either homogenization or sonication and cell debris was cleared by centrifugation for 1 h at 4 °C in a JLA 16.250 rotor at 13,000 rpm. A total of 10 mM imidazole was added to the supernatant, before being applied to a nickel-nitrilotriacetic acid (Ni-NTA) column and incubated at 4 °C for 1 h. The column was washed with 50mM Tris/HCl pH 7.5/7.8, 500 mM NaCl, 2 mM DTT, and increasing concentrations of imidazole. The protein was eluted with 100–200 mM imidazole, 50 mM Tris/HCl pH 7.5/7.8, 500 mM NaCl, and 2 mM DTT. The protein was digested with TEV protease overnight at 4 °C in dialysis buffer (50 mM Tris/HCl pH 7.5/7.8, 500 mM NaCl, and 2 mM DTT), before being applied to an Ni-NTA column again to remove the protease and affinity tags. The cleaved protein was then concentrated and purified further using size exclusion chromatography (SEC) equilibrated with 20 mM Tris/HCl pH 7.5/7.8, 200 mM NaCl and 1 mM TCEP. The SEC fractions were analyzed by SDS-PAGE before being pooled and concentrated (10 kDa cut-off).

### 2.2. Crystallization and Structure Determination, Molecular Modeling

For crystallization of the mutated (see Section 3.1), TEV-cleaved Dio2-71-262, crystals were set up in a variety of screens at 4 °C using 0.2 µL of protein solution (20 mg/mL) with 0.2 µL of reservoir solution. Within 3 days crystals appeared; some of the conditions were optimized further but some crystals could be mounted directly from the screen plate. The structure was obtained from a crystal grown in screen PACT (Quiagen, Hilden, Germany); 0.1 M SPG buffer pH 7.0 and 25% (*w*/*v*) PEG 1500. The crystal was mounted and flash-cooled in liquid nitrogen with no additional cryo-solution. Diffraction datasets were collected at 100 K at BESSY II beamline 14.1 [31].

Data processing was performed with XDSAPP [32], a graphical interface of XDS [33], for indexing, integration, scaling, sorting, and data conversion. The molecular replacement for the Dio2 structure was calculated with Phaser [34] from the Phenix Suite [35,36] using the published Dio3 structure as a search model (pdb code: 4TR3). The structure was refined with Refine [37,38] from the Phenix suite. Coot [39] was used for model building after each cycle of refinement until the R and R_free_ could no longer be improved upon. Graphical representations of the structure were generated using PyMol (v2.5.4; Schrödinger Inc., New York, NY, USA) combined with DSSP.

A comparison of the Dio3^cat^ and Dio2^cat^ structures was performed with PyMod [40], a PyMol plugin, and a homology model for Dio1 was then generated with these templates by using MODELLER [41] and PyMod. Catalytic core dimer modeling with experimental restraints was performed with Haddock [42], and modeling of full-length dimers with AlphaFold2 [43] and ColabFold [44].

### 2.3. Blue Native PAGE

Blue Native PAGE was performed with a 5% stacking gel and a 12.5% resolving gel. Once the gel had polymerized, the protein samples were mixed with 10% (*v*/*v*) glycerol and applied to the gel. A cathode buffer was added to the inner chamber of the Mighty small II SE250 mini electrophoresis system (Hoefer, Bridgewater, MA, USA) and the anode buffer was added to the lower, outer chamber. Electrophoresis was performed at 4 °C for 15 min at 150 V before increasing the voltage to 200 V for 2–4 h. The gels were destained in desalted H_2_O.

### 2.4. Cross Linking and Mass Spectrometry Analysis

Proteins were transferred into 20 mM HEPES pH 7.5, 200 mM NaCl, and 1 mM TCEP via SEC. The cross-linker stock solution was prepared as 100 mM DSSO in HEPES buffer. In total volumes of 15–30 µL, proteins were cross-linked at room temperature for 30 min or 1 h. Reactions were stopped through the addition of 1 µL of Tris/HCl pH 8.0. Reduction and alkylation of the protein samples were performed according to a set protocol [45]. The samples were then digested in 25 mM ammonium bicarbonate with 1 µL trypsin solution (200 ng trypsin in 0.01% formic acid). Samples were incubated at 4 °C for 30 min to 2 h, and then placed in an incubator overnight at 37 °C before being spun down the next day at 10,000 rpm for 10 min. The supernatant was injected into a pre-column for desalting and then a C18 column for peptide separation. Peptides were analyzed on an LTQ mass spectrometer using MS^3^ fragmentation and data analyses were performed using Byonics and following the cross-linked peptides identification scheme proposed by Kao et al. [46].

### 2.5. Activity Assays

Thyronine deiodination activity was assayed in a total volume of 100 μL, containing 100 mM KPO_4_ at pH 8.0, 0–20 µg of Dio3 enzyme, and 1 µM T_4_ substrate in the presence or absence of 20 mM DTT as the thiol cofactor. After incubation between 0 and 3 h at 37 °C, reactions were stopped through the addition of 10 µL of 100% acetic acid. Samples were analyzed as duplicates by mass spectrometry for the product rT_3_, as described with minor modifications: two 95 µL acetic acid-stopped aliquots of the reaction mixture were diluted to 400 µL with 305 µL PBS for liquid–liquid extraction of thyroid hormone metabolites [47].

### 2.6. Recombinant Production of Human Dio2 Protein and Radioactive Dio2 Assay

Cloning of C-terminally Flag-tagged full-length human Dio2 mutants was carried out as described in [8] using the following primers: DIO2-U133C-fwd 5′ gtggtcaactttggctcagccacttgtcctcctttcac 3′, DIO2-Sec133Cys-rev 5′ gtgaaaggaggacaagtggctgagccaaagttgaccac 3′ and DIO2-Ala131Cys-Sec133Cys-fwd 5′ gtggtcaactttggctcatgcacttgtcctcctttcac 3′, DIO2-Ala131Cys-Sec133Cys-rev 5′ gtgaaaggaggacaagtgcatgagccaaagttgaccac 3′. Recombinant expression and enrichment of the Dio2 mutants as well as radioactive deiodinase assays were performed as described previously [8]. The reducing systems for deiodinase assays consisted of either 20 mM DTT, 10 mM GSH, 3 µM Trx + 0.1 µM TrxR + 200 µM NADPH, or 1 µM Grx + 1 mM GSH.

## 3. Results and Discussion

### 3.1. Crystal Structure of the Mouse Dio2 Catalytic Domain

To explore the molecular basis of deiodinase isoform differences, we solved a crystal structure of the catalytic domain of *Mus musculus* Dio2 (mDio2^cat^; residues 71–262). The catalytic Sec^130^ was replaced with cysteine to simplify bacterial overexpression. Initial crystallization trials were not successful, and we thus employed a “surface entropy reduction” (SER) approach [48] to improve the likelihood of crystal formation. Two lysine residues, Lys^180^-Lys^181^, were predicted as the best SER candidates based on sequence analyses with the SERp server [48] and consistent with the structure of the homolog mDio3^cat^ (likely surface position) [6] and were thus mutated to Ala. The protein mDio2^cat^-(Lys^180^Ala-Lys^181^Ala; mDio2^cat^-AlaAla) indeed formed well-diffracting crystals and the structure of mDio2^cat^-AlaAla (from now on referred to as mDio2^cat^) could be solved and refined at a resolution of 1.1 Å to *R/R_free_* values of 14.5% and 16.9%, respectively (Figure 1B; Table 1).

The mDio2^cat^ structure comprises 183 residues (Figure 1B), covering an N-terminal Gly as a cloning artifact, a small part of the linker to the transmembrane region (71–76), and the complete catalytic core (77–261), except for the C-terminal Arg^262^ and residues 94–102, which are not defined by electron density. All residues of the structure are within favored (96.5%) or allowed regions (2.9%) of the Ramachandran plot, except for Phe^224^, which is well defined by electron density. mDio2^cat^ features a Trx/Prx fold with deiodinase-specific modifications and insertions (Figure 1B,C) and high similarity in the overall structure to mDio3^cat^, confirming the proposed conservation of a Trx/Prx-related fold among the Dio isoforms [6,7]. The mDio2^cat^ core is made up of a seven-stranded mixed β-sheet, surrounded by four α-helices (Figure 1B,C). The basic Trx fold provides β-strands β1–4, and the sheet they form is extended by a single strand, βD, from the deiodinase-specific insertion that further comprises an α-helix αD and the extended loop-D (residues 161–188), which protrudes out of the globular structure. The sheet is further extended by an N-terminal Prx-like module consisting of two β-strands, βN1/2, followed by a small 3_10_-helix, ϴ1. The N-terminal linker residues, and likely also the missing loop 94–102, point away from the globular core domain.

### 3.2. The Dio2-Specific Insertions and PRDX-Motif

**Figure 2:**
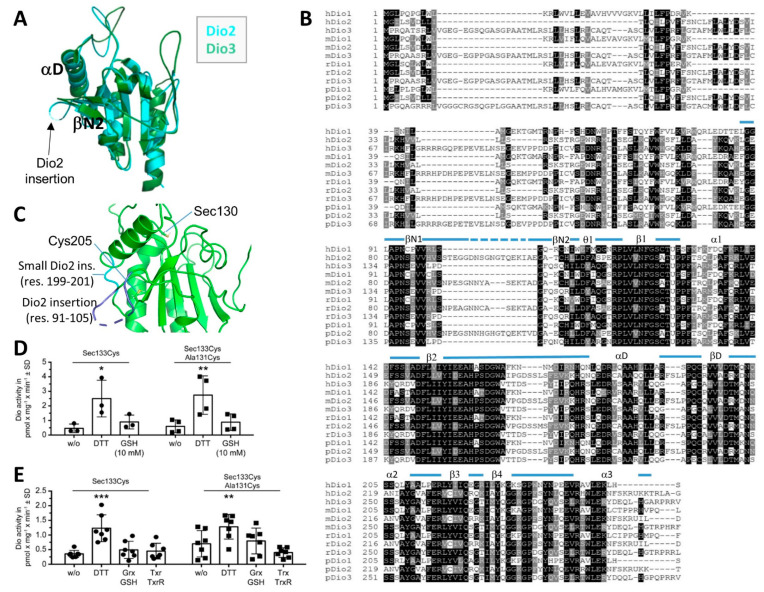
Dio2-specific structural and catalytic features. (**A**) Overlay of the crystal structures of the catalytic domains from mouse Dio2 (cyan) and Dio3 (green), respectively. The Dio2-specific insertion is indicated. (**B**) Sequence alignment for Dio1–3 catalytic domains from human (h), mouse (m), rat (r), and pig (p). The secondary structure elements of mDio2 are illustrated on top (dotted line: not defined by electron density). (**C**) Crystal structure of Dio2 with the catalytic residue Cys130 (labeled as Sec130), the distal Cys205, and two Dio2-specific insertions indicated (blue). (**D**) Specific activity of enriched human Dio2(Sec133Cys) after recombinant expression in insect cells; 10 mM GSH did not support deiodination of 125I-rT_3_, while DTT was able to serve as a reductant. Introduction of a proximal Cys^131^ neither increased Dio2 activity with DTT nor allowed GSH to serve as a reductant. N = 3–4 independent experiments. * *p* < 0.05, ** *p* < 0.01. Two-way ANOVA followed by Dunnett’s *t* test. (**E**) Specific activity of enriched human Dio2(Sec133Cys) and Sec133Cys/Ala131Cys recombinantly expressed in insect cells. Neither Grx nor Trx supported deiodination of 125I-rT_3_, while DTT served as a reductant. N = 7 independent experiments. ** *p* < 0.01, *** *p* < 0.001. Two-way ANOVA followed by Dunnett’s *t* test.

An overlay of our mDio2^cat^ structure with mDio3^cat^ (PDB ID 4TR3; Figure 2A) confirms that the overall structures of these Dio isoforms are highly similar (also at the Dio2 LysLys->AlaAla mutation site, which enabled a crystal contact; Supplementary Figure S1A,B), including most of the modifications to the basic Trx fold. A major difference between Dio2 and other isoforms, however, is a 15-residue (91–105) “Dio2-insertion” in the N-terminal region of the catalytic core (Figure 2B). The structure reveals that the insertion is located between βN1 and βN2 in the Prx-like module (Figure 1B,C and Figure 2A). It extends toward αD and then points away from the globular domain. The corresponding region in Dio3—and, based on the homology, also in Dio1 (Figure 2B)—forms a tight turn at the core’s surface (Figure 2A). Interestingly, there is no electron density for nine central residues of the Dio2-insertion (94–102), indicating conformational heterogeneity or high flexibility. The Dio2-insertion has previously been dubbed the ”destruction loop” [49], because it appears to be essential for the Dio2-specific process of ubiquitination and subsequent proteasomal degradation induced by deiodination of a T_4_ substrate molecule [16,49,50]. Ubiquitination also appears to decrease directly Dio2 activity via a conformational change [30]. The mDio2^cat^ structure shows that the Dio2-insertion (residues 91–105) and its conserved Glu-Lys-Thr/Ile-Ala/Val motif is surface exposed and positioned close to the so-called “distal Cys”, Cys^205^ in Dio2 (Figure 2C), which in related proteins appears to support re-reduction in the active site Sec (see also below) [49]. Strikingly, Cys^205^ is flanked by a second Dio2-specific insertion of three residues (199–201 in mDio2; Figure 2B,C). An additional Pro there (Pro202; Figure 1A) and pronounced interactions of this small insertion with the larger Dio2-insertion (Figure 2B,C) likely reduce the ability to adapt local conformation to the needs of disulfide formation and interactions with redox ligands. These features likely hinder re-reduction in Dio2, consistent with data indicating that Dio2 undergoes no or only very inefficient re-reduction (see below) [16]. Further, it is tempting to speculate that the Dio2 redox state will influence, through the interplay of these two Dio2-specific insertions, conformation and/or flexibility of the destruction sequence and thus render this surface region of the catalytic core sensitive to T_4_ deiodination. A previous model for the Dio2 dimer interaction with E3 suggested two ligase units attached at opposite poles of the core dimer [49], which would indeed correspond to the exposed destruction sequence location (see also below for details of the dimer architecture). Such an interaction thus could influence the Dio2 dimer orientation and expose the sites of ubiquitination, Lys^234^ and Lys^241^ [30], which we find are located close to the dimerization surface (see below). The ubiquitination site Lys^241^ could also influence the active site directly through an interaction with the Phe^224^ residue in the substrate site, analogous to the corresponding residue Arg^275^ in Dio3, or by causing a rearrangement of β3/β4 located right before Lys^241^ and comprising Phe^224^ right before β3.

Comparison of the mDio2^cat^ crystal structure to other thiol redox protein structures from the PDB reveals that an insertion corresponding to the Dio2-insertion exists in the peroxidases of the alkyl hydroxyperoxide reductase 1 (Ahp1) family, which are also inhibited through a protein-attaching modification (urmylation) [51]. In these proteins, however, the sequence insertion contains the “resolving Cys” (Cys^R^) that crosslinks with the catalytic “peroxidatic Cys” (Cys^P^) of a second monomer bound via the A-type Prx interface (see below) [52,53], indicating that this structural element developed into a different function in Dio2. Interestingly, Ahp1 proteins also contain the second insertion, corresponding to mDio2-199–201, to form a pronounced interaction between the larger “Dio2-specific”-like insertion and the loop next to the distal Cys [53]. The structural arrangement thus appears conserved and relevant to the functions of Ahp1 proteins and type 2 deiodinases, but future studies will have to reveal the details of their functional roles.

While inactivation and subsequent proteolytic degradation of Dio2 after exposure to substrate (e.g., T_4_ or rT_3_) are well established [5,16], it is less clear whether oxidized Dio2 can undergo an efficient reduction step as part of a catalytic cycle in vivo. In vitro, Dio2 activity is readily measured using the non-physiological reductant DTT, but to our knowledge, no physiological reducing co-factor of Dio2 has been identified—unlike GSH, Grx, or Trx as co-factors for Dio1 and Dio3 [6,8,15]. We previously showed that Dio3 and Dio1 can be reduced with DTT even in the absence of the two conserved Cys residues, which were termed the proximal and the distal Cys, respectively [6,8]. These results are in line with previous work, in particular very similar experiments from Croteau [15], and might indicate that Dio2 catalytic cycling occurs only under artificial in vitro conditions with DTT as a reductant. In Dio2, in addition to its sequence insertions, the proximal Cys (position 128 in mouse, position 131 in human) is normally replaced by Ala. Here, we tested whether introducing a proximal Cys at position 131 in human Dio2 could enhance its activity. We expressed human Dio2-Sec133Cys in the insect cell system described before [8] and incubated the enriched enzyme with ^125^I-rT_3_ for deiodinase activity measurements (Figure 2D). While the enzyme was active with DTT as a reducing co-factor, it was not reduced by 10 mM GSH. A mutant containing in addition a proximal Cys at position 131 (Ala131Cys) exhibited the same specific activity as the Sec133Cys enzyme with DTT, but it remained inactive with 10 mM GSH (Figure 2D). We next tested reduced Trx or Grx as reducing co-factors, but neither Dio2-Sec133Cys nor the mutant with the additional proximal Cys showed an increase in activity in response to Grx or Trx addition (Figure 2E). This finding suggests that Dio2 is indeed not designed to be re-reduced efficiently by a physiological redox cofactor and may act as a single-turnover enzyme. Rather, either solely the oxidation of Sec^130^ or the formation of a selenylsulfide intermediate between the catalytic Sec^130^ and the distal Cys^205^ may induce the conformational change that enhances the ubiquitination of substrate-exposed Dio2. We can conclude that while the lack of a proximal Cys appears to contribute to the unique behavior of Dio2, it is not sufficient and the Dio2 insertions likely are also relevant factors.

The third Dio2-specific insertion comprises three residues within the tip of the Dio-insertion loop, folded on top of the kinked helix α2. Insertion and loop likely contribute to substrate binding and positioning, and thereby to the isoform’s region-selectivity, and will thus be discussed in the next section.

### 3.3. Isoform-Specific Regioselectivity for Thyronine Deiodination

**Figure 3:**
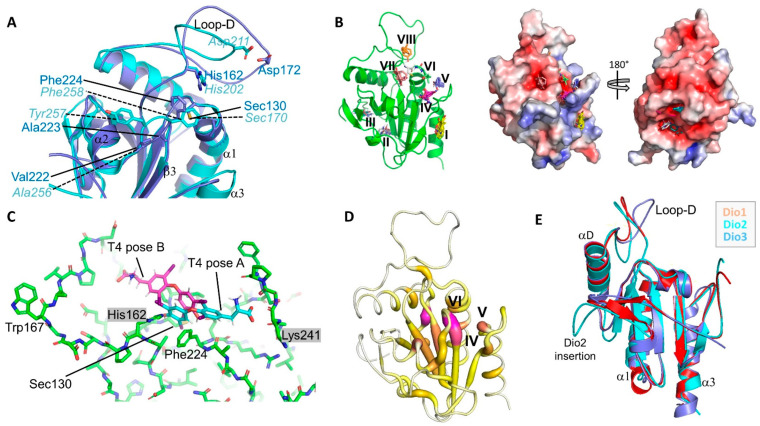
The Dio substrate binding site. (**A**) Overlay of the active site regions of Dio2 (blue) and Dio3 (cyan). Key residues and secondary structure elements are labeled. (**B**) Preferred interaction sites for small molecule probes on Dio2, identified with *FTmap*. Left: Cartoon presentation with sites numbered. Right: Front and back view of the Dio2 surface colored according to electrostatic potential (calculated with APBS; red: negative potential, blue: positive). (**C**) Active site region of Dio2, with the ligand T_4_ modeled manually in two orientations: pose A with the carboxylate in site IV (see panel **B**), and pose B with the carboxylate in site VIII. The residues corresponding to the previously suggested mDio3 substrate clamp are indicated by gray boxes. (**D**) Ribbon presentation of Dio2 colored according to the CryptoSite score (scale from 0 to 25). Highest scores are shown in magenta/fat ribbon, lowest scores in white/thin ribbon. (**E**) Overlay of the crystal structures of mDio2 (cyan) and mDio3 (blue) catalytic domains and a homology model of the mDio1 catalytic domain (red).

Comparing the crystal structures of mDio2^cat^ and mDio3^cat^ revealed subtle differences in the active site centers (Figure 3A). Most residues in this region (on the α2/β3 loop) are highly conserved amongst the isoforms, signifying a role in ligand binding and catalysis. However, the identity and positioning of some residues vary, indicating a likely contribution to the difference in regio-specificities of the isoenzymes. In the Dio3^cat^ structure, the active site, and, in particular, the Sec^170^, is shielded by a strained conformation of Phe^258^ from the α2/β3 loop and it was speculated that substrate binding or Dio3 dimerization could release this strain and allow access to the active site Sec [5]. Strikingly, our mDio2cat crystal structure also featured a single Ramachandran plot outlier, Phe224, which is well defined by electron density and corresponds to mDio3-Phe258 (Figure 3A). This residue thus appears again to assume a strained conformation. The two residues are overlaid yet orient their side chains slightly differently. Importantly, the different side-chain orientations support a different backbone conformation for the two preceding residues (mDio2-Val^222^Ala^223^ vs. mDio3-Ala^256^Tyr^257^), which is needed for accommodating the larger mDio2-Val side-chain compared to the mDio3-Ala. These differences loosen the coverage of the catalytic Sec/Cys by mDio2-Phe^224^ and widen the gap between this backbone region and the end of β2 and the conserved His (mDio2-His^162^) implicated in substrate binding [6].

Analyzing the mDio2^cat^ structure for potential ligand binding pockets using the FTmap algorithm that probes the entire protein surface for small organic molecule binding hot spots (https://ftmap.bu.edu; accessed on 23 February 2022) [54] yielded eight candidate sites. Three sites located too far from the active site for direct involvement in substrate binding (Figure 3B): The shallow site (I) is preferred by hydrophobic fragments and likely contributes to dimerization (see below), and sites (II) and (III) are pockets in the negatively charged bottom back of the monomer that preferably accommodate nitrogen-containing fragments. Five sites could possibly contribute to TH binding: (IV) and (V) form the crevice between the β4/α3-loop and the catalytic center, and (VI) is an invagination from the crevice toward the catalytic Sec/Cys, flanked by Phe^224^ and Pro^132^ (Figure 3B); site (VII) locates on the other side of Phe^224^ and thus is currently shielded from the catalytic Sec; and site (VIII) is a cavity formed by the D-insertion and covered by its Ω-loop. Interestingly, the alkyl chains of alkyl peroxidase ligands can exploit a site corresponding to site (IV) [52], which is lined by Lys^241^ (Figure 3C). Previously, the corresponding mDio3 residue Arg^275^ was proposed to act as clamp binding the substrate carboxylate and/or to close the active site upon substrate binding to a—partly cryptic–site between mDio3-Arg^275^ and His^202^ (corresponding to mDio2-His^162^; Figure 3C) [5,6,9]. While the Dio3-Arg^275^ is oriented toward the active center, directly closing site (IV), the Dio2-Lys^241^ is positioned perpendicular to this direction, creating the site (IV)/(V) crevice by forming a wall more distant from the catalytic site (Figure 3C). Thereby, an interaction of the substrate α-amino carboxylate with this residue allows the ligand to stick out further from the active site than in Dio3 and thus to position the outer ring, rather than the inner ring, next to the catalytic Sec (Figure 3C, “pose A”). Dio2-Arg^226^ forms the floor of this site and should support the positioning of the substrate carboxylate, while Glu^225^ might support substrate positioning through binding of the α-amino group. Strikingly, when we analyzed Dio2^cat^ for potential occluded T_4_ binding sites using the CryptoSite support vector machine algorithm that scores sites based on sequence conservation, molecular dynamics calculations, and a likelihood of fragment binding (https://modbase.compbio.ucsf.edu/cryptosite; accessed on 23 February 2022) [55], we obtained potential binding sites well matching the FTmap results and fitting to the T_4_ binding mode “pose A” described above (Figure 3C,D). The branched crevice formed by sites (IV), (V), and (VI) is indicated as a major candidate for an adaptable site, together with a negatively charged back side pocket next to sites II/III whose functional relevance remains unclear. Putting T_4_ into this site allows to position the outer ring with the reaction site iodine next to Sec^130^, the inner ring in site (IV), and the α-amino carboxylate in the site (V)/(VI) crevice.

The pronounced sequence conservation among Dio isoforms allowed us to use the mDio2^cat^ and mDio3^cat^ crystal structures and our alignment for generating a reliable homology model of the mouse Dio1 catalytic domain (mDio1^cat^; Figure 3E; Supplementary Figure S1C). We then used this model to include Dio1 in our structure analyses. The comparison illustrates that mDio1^cat^ is highly similar to the generic Dio catalytic core structure defined by the two crystal structures, and that it closely resembles, in particular, mDio3^cat^. Interestingly, the Sec-shielding, strained Phe conserved in Dio2 and Dio3 (Dio2-Phe^224^, Dio3-Phe^258^) is replaced by a Pro conserved in Dio1 (mDio1-Pro^213^). This replacement opens the cleft toward the catalytic Sec even further than in Dio2 (see above) and provides ample—and due to its rigidity also stable—space for the Dio1 TH substrates (Figure 3E; Supplementary Figure S1C). This Dio1 feature, together with the conserved replacement of the second Pro in the Dio2/Dio3 Pro-Pro motif (mDio2-Pro^131^-Pro^132^) to Ser (mDio1-Ser^128^), seems to result in a wider and more flexible substrate site and thus could explain the lower substrate affinity of Dio1 [10] and the larger variation of TH substrates accepted by this isoform, such as TH-sulfates and the TH-related acetic acid metabolites, such as tetraiodothyroacetic acid (TETRAC) [56]. The wider Dio1 side could also explain how it can accommodate either a Dio2-like or Dio3-like substrate pose required for deiodination at the different Dio1 target positions (inner vs. outer ring; Figure 1A and Figure 3C) [1]. In principle, it could also be envisioned that the outer ring iodine could be placed in the Dio1 active site through a 180° rotated binding mode, pointing the carboxylate toward loop-D and sites VI and VIII (Figure 3C, “pose B”). Such a binding mode might explain why Dio1 efficiently deiodinates TH-sulfates, which would occupy the carboxylate site with the sulfate group. Since we have no experimental evidence for such a reversed binding mode, however, we currently assume that Dio1 binds substrates in the same orientation as Dio2 and Dio3. The Dio1 variability in regio selectivity within a conserved generic TH binding mode is well supported by the architecture of the putative α-carboxylate site: the Arg forming the binding site floor and the Glu contacting the amino group are also conserved in Dio1 (Dio2-Glu^225^Arg^226^/Dio1-Glu^214^Arg^215^; Supplementary Figure S1C). The conserved Dio2-Lys, respectively Dio3-Arg, fine-tuning the carboxylate position and thereby the ring positions in the substrate cleft, however, is replaced in mDio1 by Ala^230^. The Ala is replaced by Ser or Pro in some Dio1 sequences from other species (Supplementary Figure S2), consistent with this residue not contributing significant substrate interactions in this isoform. The Dio1 replacement is coupled with the introduction of a neighboring Lys, yielding a conserved Lys-Ala/Pro/Ser motif in Dio1 (mDio1-Lys^229^Ala^230^) that replaces the conserved Gly-Lys in Dio2 and Gly-Arg in Dio3. The Lys in the Dio1 motif might help to keep the general electrostatics of the binding site region constant yet move the charged side chain too far away from the carboxylate site for direct interaction, facilitating substrate binding in two states that differ in how deep the TH is inserted in the substrate cleft.

The large Ω-loop within the Dio insertion, loop-D, contains highly conserved residues at its edges, and some of them were suggested to contribute to forming the substrate site and/or to a catalytic proton shuttle connecting the active site and protein surface [6,9]. The loop center shows small differences in sequence and length between isoforms but is again highly conserved within isoforms, and it is tempting to speculate that it contributes to substrate binding and the isoform differences in regio specificity. Molecular dynamics studies on Dio3 indicated different loop-D conformational states, including “closed” states that could cover the top of the ligand binding site [9]. Thereby, in addition to a proposed interaction of TH with the conserved His (Dio3-His^202^) in the loop-D edge—hydrogen bond to 4′-OH [6] or packing against the outer ring [9]—a halogen bond interaction of the central loop-D residue Dio3-Asp^211^ to the substrate 3′-I was formed. Compared to Dio3, the Dio2 loop-D is slightly elongated (three more residues) and in a more open state in the mDio2^cat^ structure (Figure 2A and Figure 3A). High B-factors suggest conformational heterogeneity, likely due to increased flexibility as indicated by the observation that the backbone density is fuzzy but a dominant chain conformation is clearly visible. Such an increased flexibility would be consistent with the proposed closure movement. Strikingly, such a closure would allow the binding hot spot site (VIII), formed by the backbone of the Sec loop (loop β1/α1) and the loop-D center Dio2-Pro^170^ to Dio2-Glu^178^ (Figure 3A), to complete the TH binding site by covering the outer ring. Thereby, Dio2-Asp^172^, the Dio2 residue corresponding to the suggested halogen bonding Dio3-Asp^211^, could form a similar halogen bond interaction to the enzyme’s substrate 3′-I. Due to the slight extension of loop-D in Dio2, this interaction would support the binding mode described above, with the TH ligand slightly shifted toward the carboxylate pocket compared to Dio3, positioning the outer ring rather than the inner ring next to the Sec for the catalytic attack. The absence of this interaction in Dio1 would be compatible with a more variable positioning of the substrate, allowing for both inner and outer ring deiodination.

The residues of the loop-D edges conserved among all Dio isoforms appear to support Dio catalysis through several functions. The conserved residues corresponding to Dio2-Glu^160^, -His^162^, and -His^182^ were proposed to form a catalytic proton relay system together with Ser^127^, Thr^129^, and Tyr^157^, and His^202^ was implicated in substrate 4′-OH binding [6]. This hydrogen bond network is indeed also formed in the mDio2^cat^ structure (Figure 3A), and Dio3 MD calculations indicated that the network remains mostly stable during the transition between different loop-D states [9]. The MD calculations suggested, however, for Dio3-His^202^—corresponding to Dio2-His^162^—a more passive function than the previously proposed polar interaction with the substrate 4′-OH, as a stacking partner for the conserved Dio3-Trp^207^ (Dio2-Trp^167^). Such a Dio2-His^162^/Trp^167^ interaction in Dio2 seems plausible, given the exposure of one side of the His^162^ plane, and would drive loop-D toward closure. It exploits Glu^160^ as a buried anchor fixing the position of the loop starting point [9] and the conserved Pro^163^ as a kink helper. The interaction would also fine-tune the position of His^162^, which can still interact with the ligand outer ring. Based on its position, we assume that it either slightly swings around to form a hydrogen bond or packs parallel and/or forms an electrostatic interaction with the 4′ group. While further mutational studies and a substrate-bound co-crystal structure of the enzyme will be required to fully understand Dio/TH binding, His^162^ clearly appears to act as a key residue contributing to several functions.

### 3.4. Deiodinase Dimerization

**Figure 4:**
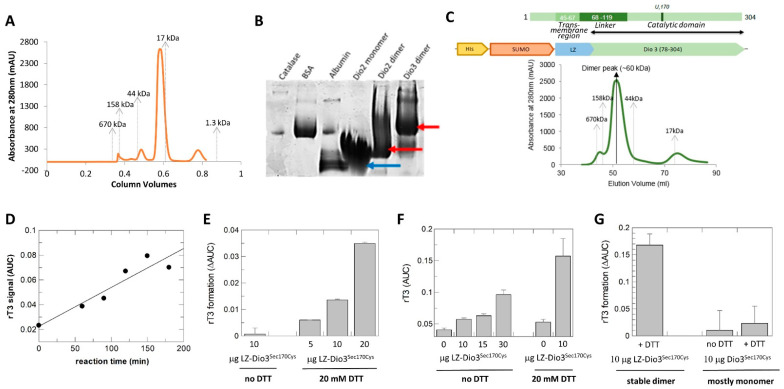
Dio dimerization and activity. (**A**) SEC profile of mDio2-71-262. Elution positions of molecular weight standards are indicated. (**B**) Blue native PAGE of Dio2 samples eluted from SEC at the size corresponding to monomer or dimer, respectively. For comparison, catalase (240 kD), bovine serum albumin (67 kD), and chicken egg albumin (45 kD) are shown. The Dio3 dimer represents LZ-mDio3 (see panel **C**). Arrows indicate main positions of the Dio bands. (**C**) Engineering of a stable mDio3-cat dimer. Schemes of the mDio3 domains (black double-arrow: length of mDio2^cat^ construct) and the fusion construct with a dimerizing Leu zipper (LZ). Below the schemes, a SEC profile of the purified LZ-mDio3-cat construct is shown. Elution positions of molecular weight standards are indicated. (**D**) Time-dependent increase in rT_3_ formed from T_4_ by LZ-mDio3-cat in presence of DTT. (**E**) Increase in rT_3_ formation in presence of increasing enzyme amounts in presence of DTT. (**F**) Enzyme-dependent increase in product rT_3_ in absence of DTT (left), and substantial increase in product formation in presence of the redox cofactor (right). (**G**) Comparison of rT_3_ formation by the stable LZ-mDio-cat dimer (left) and the mostly monomeric mDio3-cat (right).

Previous studies indicated that homodimerization is required for deiodinase activity and while the N-terminal transmembrane region is essential for stable dimer formation, the catalytic core itself already shows weak homodimerization [27,28,29,30]. In this work and the previous Dio3^cat^ study [6], partial dimerization was indeed observed with N-terminally truncated recombinant core constructs but the monomer was dominant in solution. Purification of Dio2-71-262, lacking the N-terminal 70 amino acids including linker and transmembrane anchor, yielded a dominant monomer and only small amounts of a potential dimer in the final size exclusion chromatography (SEC) step (Figure 4A). The latter eluted at a volume consistent with the dimer mass and being twice the size of the monomeric species. SEC-MALS (Multi Angle Light Scattering) analysis confirmed the two species to be Dio2 monomer and dimer, respectively (Supplementary Figure S3A). Incubation of either isolated species for 24 h and re-analysis in SEC experiments showed only a slight redistribution between monomeric and dimeric species (Supplementary Figure S3B), indicating that they are rather stable and that the transition between them is slow, which might indicate that major conformational changes are required. Consistent with the SEC-MALS results, Blue Native PAGE analysis identified both, Dio2 monomer and dimer, in both samples (Figure 4B). The monomeric species showed a strong band at the monomer size but a significant smear to the dimer position, and the dimeric species showed a major band at the dimer as well as the monomer position with a smear in between. These results confirm with highly purified proteins the model from cellular experiments that the Dio catalytic core allows dimerization but requires support from the N-terminal linker and/or transmembrane region for full stability [27,28]. Strikingly, a longer construct Dio2-49-262 yielded mostly dimer (but limited yields, and it failed to crystallize; Supplementary Figure S3C), confirming that the linker region between transmembrane-region and catalytic core support formation of stable dimers, but that membrane attachment is not required for dimer formation. Our results further show that the transition between monomer and dimer is slow, possibly indicating that larger conformational changes are involved.

We next set out to ask in our highly purified in vitro system whether dimerization of the Dio catalytic domain is indeed required for activity and whether the membrane anchor is essential for the formation of active dimers. We chose Dio3 for this analysis since activity could easier be analyzed for Dio3 than Dio2, while our structure comparison showed that the basic features of the catalytic cores are conserved and results thus should be applicable to all three Dio isoforms. For a reliable comparison of monomeric and dimeric species, we used the dominantly monomeric construct mDio3^cat^ (mDio3-78-304^Sec170Cys^) and generated a fully stable dimeric version through N-terminal fusion with a Leucine Zipper (LZ) dimerization module from *A. thaliana* Hy5 (Figure 4C). The enforced LZ-mDio3^cat^ dimer was tested with T_4_ as the substrate in a mass spectrometry-based activity assay. Even though the catalytic Sec was replaced by Cys in all our constructs to simplify bacterial expression, which is known to lower Dio activity ~1000-fold [57], the dimeric LZ-mDio3^cat^ was able to catalyze rT_3_ formation. Product formation increased over time (Figure 4D) and could be accelerated by increasing the enzyme amount (Figure 4E). Activity in the absence of DTT was low but roughly proportional to the enzyme amount, consistent with a single-turnover reaction (Figure 4F), and in all settings it vastly increased in the presence of DTT (Figure 4E,F). LZ-mDio3^cat^ thus is a catalytically active Dio enzyme, able to bind T_4_ substrate and to catalyze turnover to rT_3_. Strikingly, equal amounts of mDio3^cat^, which is identical to LZ-mDio3^cat^—with a catalytic Cys—except for the LZ module and thus mostly monomeric, showed no statistically significant activity (Figure 4G), supporting the conclusion that dimerization is required for Dio activity.

**Figure 5:**
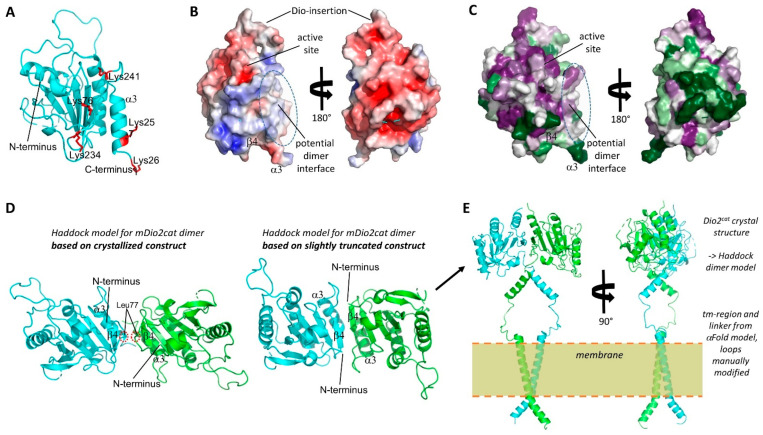
Dio dimer architecture. (**A**) Crystal structure of mDio2 catalytic domain. The Lys residues identified through cross-linking MS to contribute to dimerization are shown in red and labeled. (**B**) Front and back view of the surface of the mDio2-cat crystal structure colored according to the electrostatic potential (calculated with APBS; red: negative potential, blue: positive). Key regions are indicated. (**C**) Front and back view of the surface of the mDio2-cat crystal structure colored according to sequence conservation (magenta: high conservation; green: low conservation). Key regions are indicated. (**D**) mDio2-cat dimers generated by Haddock based on the mDio2-cat crystal structure and cross-linking results. Using the complete crystal structure results in an anti-parallel dimer with a small interface formed by the N-terminal linker fragments sandwiched between the monomers’ β-sheet (left). Slightly truncating the N-terminal linker allows a similar association, but with improved interface through direct association of the sheets via β4. (**E**) Front and side view of a model for full-length mDio. The N-terminal trans-membrane helix and the linker were modeled with AlphaFold2, and the linker helix orientation manually adjusted to allow fusion to the N-termini to the optimized catalytic domain dimer (panel **D**, right).

The architecture of the Dio dimer and how it supports catalytic activity is poorly understood [5,6]. To investigate the dimer architecture, we first analyzed the protein/protein interactions in the mDio2^cat^ crystals with the PISA (Proteins, Interfaces, Structures, and Assemblies) server [58]. All interactions appeared to be crystal contacts causing arrangements that would be unstable in solution and generate unlikely, highly asymmetric dimers with unsaturated interaction interfaces (Supplementary Table S1). This conclusion is consistent with the fact that crystals were only obtained with the dominant monomer fraction of the mDio2-71-262 construct. We thus employed a cross-linking approach to obtain experimental data on the Dio dimer arrangement. Incubation of either the mDio2-71-262 monomeric or dimeric species with the cross-linking reagent disuccinimidyl sulfoxide (DSSO) followed by tryptic digest and mass spectrometry analysis identified Lys^76^, Lys^234^, Lys^257^, Lys^261^, and Lys^241^ as cross-linking sites. Each of these Lys residues was found to have multiple links to other Lys residues from this set, and as expected, the number of crosslinks that could be identified was higher for the dimer species (Table 2). Lys^76^ is part of the N-terminal linker sequence, while all other identified Lys residues are located in the β4α2 motif of the catalytic core. Strikingly, mapping these residues on the Dio2^cat^ structure shows that they encircle a surface patch comprising the edge of the β-sheet, the shielding helices, and the N-terminal linker, suggesting this surface as a Dio dimerization interface (Figure 5A). The identified surface is rather flat, similar to typical protein/protein-interaction interfaces, and lowers the demand for shape complementarity (Figure 5B).

To analyze whether other surface properties would be consistent with a dimerization function, we first calculated the electrostatic surface potential. The monomer shows overall the expected significantly charged surface potential, mostly with a negative charge (Figure 5B). The putative dimerization interface, however, shows weaker polarity, with a mix of slightly positive and negative patches as well as hydrophobic regions, consistent with a dimerization function (Figure 5B). We further analyzed the conservation of this Dio2 surface area. Calculating position-specific conservation scores from an alignment of 45 Dio1, Dio2, and Dio3 sequences (Supplementary Figure S2) using “ConSurf” [59] and mapping it on the monomer surface showed that, as expected, the catalytic site region around Sec130 is highly conserved (Figure 5C). Strikingly, the potential dimerization interface shows a large area of significant conservation as well, varying from moderate to high conservation, while the remaining Dio2 surface shows mostly strong variation with only smaller patches of conservation. These results hint strongly at a conserved function for this surface area, supporting a role as a dimerization interface. Noteworthy, the largest Dio2 crystal contact involves the C-terminal α3 of one monomer, consistent with the observation that crystal contacts often (partially) shield protein/protein interaction interfaces.

The identified Dio2 interaction interface partly corresponds to the Dio3 dimer interface modeled previously based on a related bacterial Prx dimer, comprising the C-terminal helix α3 and a staggered association of the β-sheet edges formed by β7 [6]. Interestingly, it largely corresponds to the so-called “B-type” interface for Prx dimerization: within the Prx family, dimers are the dominant species and formed either via the “A-type” interface (“Alternate”; formed by loops preceding α2, α3, α4) or the “B-type” interface (“Beta strand”; formed through the fusion of the monomers’ β-sheets via β7) [60]. While the A-type interface is obstructed in Dio proteins by the Deiodinase insertion, the interface around β7 identified by the cross-links matches the B-type interface. B-type dimers are normally stabilized by a C-terminal extension to the Trx fold. The fact that this extension is missing in Dio enzymes might explain why they instead need the N-terminal tm/linker-region for fully stable dimers. The identified dimerization interface would allow two orientations, parallel or anti-parallel. However, dimerizing our Dio2^cat^ crystal structure using the Haddock protein/protein docking server [42] and our experimental cross-links as restraints resulted exclusively in anti-parallel arrangements for the top predictions, similar to the anti-parallel B-type dimerization of Prx proteins (Figure 5D). In contrast to Prx dimers, however, the β-sheets of the monomers would not fuse in these models, due to a small linker loop around Leu77. It would either intercalate between the sheets or force them to shift in a staggered position. The positioning of this linker loop 77–79 serves to partly shield the exposed, hydrophobic dimerization interface, and we thus removed residues 71–79 to avoid the possibly artificial steric hindrance. Docking the shortened structure, with the cross-links as restraints, indeed resulted in a Prx-like dimerization mode with fused β-sheets and exclusively anti-parallel arrangement (Figure 5D).

Noteworthy, an attempt to model full-length Dio2 dimers using AlphaFold2 [43] yielded a similar dimerization mode to our experimentally restrained Haddock model (Figure 5E), with dimerization via the C-terminal α3 and β7 with staggered sheets, albeit in a parallel orientation (Supplementary Figure S4A). We speculate that the parallel orientation is caused by the artificial packing of the N-terminus in this model, which comprised the following features shared with AlphaFold2 models we generated for Dio1 and Dio3: a long, kinked N-terminal helix α_N1_ comprising the tm region and homodimerized in a crossed orientation; one or two additional smaller helices in the linker region, packed between α_N1_ and the catalytic core; at least partial coverage of the edge formed by α3 and β7. Interestingly, among the top models were also dimers exploiting this dimerization mode with swapped C-terminal helices (Supplementary Figure S4B). Such a helix swap could be the reason why the interchange between monomer and dimer appears to be slow. We then used the generic elements of the Dio2 model, together with the experimentally determined catalytic core structure and the experimental restraints on dimerization to generate a full-length Dio2 model. The linker was extended to embed the kinked, dimerized α_N1_ in a membrane, and the Haddock model of the catalytic core dimer was attached so that direct interactions between the linker helices cover their hydrophobic surfaces (Figure 5E). In this model, all hydrophobic patches—the modeled tm- and linker-helices and the experimentally identified core patch around Lys^241^—are shielded from solvent and contribute through homomeric symmetric interactions to dimerization. Interestingly, key elements of the two substrate sites of the dimer would be involved in the dimer interaction, since the β4α2 edge and the connecting loop have been implicated by Bayse et al. [9] and our analyses (see above) to complete the binding pocket for the carboxyl end of the substrate molecule. Our results thus provide an experimentally supported molecular basis for the requirement of Dio dimerization for catalytic activity and indicate the possibility of half–of–the–sites activity, since simultaneous occupation of both active sites might be incompatible with dimer packing.

## 4. Conclusions

The crystal structure of Dio2^cat^ confirms that this isoform—and likely also Dio1—comprises the same Trx fold and Prx-like module as Dio3^cat^ and reveals the arrangements of the Dio2-specific insertions. The large Dio2-insertion extends around the monomer core, placing its tip close to the smaller Dio2-insertion and the distal Cys^205^. The selenylsulfide enzyme intermediate thus should affect the conformation of this loop, which might serve as a signal for the Dio2 ubiquitination upon catalytic substrate turnover. The conserved Dio region previously proposed to act as a proton cascade or in substrate binding was confirmed in mDIO2^cat^ to form the same arrangement as in mDio3^cat^. Comparison of these experimental structures and a Dio1 model now allowed one to propose substrate binding modes that rationalize the experimentally observed substrate preferences and regio-selectivities. Furthermore, our dimerization studies validated the original hypothesis that the Dio enzymes are functional homodimers and that the transmembrane region supports dimerization but is not crucial for catalysis or dimer formation. The experimental identification of residues at the dimerization interface allowed the generation of reliable models for Dio dimers, which also rationalize the effect of dimerization on activity. In summary, our results provide a structural basis for understanding Dio2-specific features and new insights into general properties of Dio catalysis and dimerization.

## Figures and Tables

**Table 1:** Diffraction data and refinement statistics.

	*mDio2^cat^*
*Diffraction data*	
Resolution (Å) ^a^	34.85–1.09 (1.13–1.09)
Space group	*P*32
Unit cell dimensions *a*, *b*, *c* (Å)	47.8, 47.8, 64.7
Unique reflections measured ^a^	65565 (4435)
Multiplicity ^a^	5.4 (3.6)
Completeness (%) ^a^	94.85 (64.6)
Mean I/sigma(I) ^a^	9.45 (0.4)
Wilson B-factor	14.23
*R_meas_* ^a^	0.077 (2.391)
*CC_1/2_* ^a^	0.999 (0.117)
*Refinement*	
Resolution (Å) ^a^	34.85–1.09 (1.13–1.09)
No. reflections ^a^	65,556 (4435)
*R*_work_/*R*_free_ (%) ^b^	14.5/16.9
No. non-hydrogen atoms	1657
Protein	1465
Solvent	192
RMS deviations	
bond lengths (Å)	0.015
bond angles (°)	1.5
Molprobity clash score	1.73
Average *B*-factor (Å^2^)	21.2
Protein	19.6
Solvent	33.6

^a^ Highest-resolution shell is shown in parentheses. ^b^ For R_free_ calculations, 2098 reflections were used.

**Table 2:** Dio2 Lys-Lys crosslinks.

Dio2 Lys Residue	Crosslink to Dio2 Residue	
	Using Monomer SEC Peak	Using Dimer SEC Peak
76	234, 241	76, 234, 241
234	76, 257	76, 257
241	76, 257	76, 241, 257, 261
257	234, 241	234, 241
261	---	241

## Data Availability

The atomic coordinates and diffraction data have been deposited with the worldwide Protein Data Bank (wwPDB) at https://www.wwpdb.org/ (accessed on 17 October 2024) under accession code 9H48.

## Supplementary Materials

The following supporting information can be downloaded at https://www.mdpi.com/article/10.3390/biom14111373/s1. The Supplementary Materials accompanying this publication comprise Figure S1: Dio2 LysLys->AlaAla mutation site and comparison of the active sites of mDio1, mDio2, and mDio3; Figure S2: Alignment of Dio1–3 from several species; Figure S3: Size analyses for recombinantly produced mDio2 constructs; Figure S4: Modeling of mDio2 dimers; Table S1: PISA output on mDio2^cat^ crystal interactions.
