# Supplementary material for: Structural Insights into the Iodothyronine Deiodinase 2 Catalytic Core and Deiodinase Catalysis and Dimerization
Source: Biomolecules. 2024 Oct 28;14(11):1373. doi: 10.3390/biom14111373 (PMC11592359; doi:10.3390/biom14111373)
Supplement: Supplementary file 1 [file biomolecules-14-01373-s001.zip › biomolecules-3219333-supplementary.pdf]

Supplementary Materials

Structural insights into the iodothyronine deiodinase 2 catalytic core and deiodinase catalysis and dimerization

Holly Towell[1], Doreen Braun[2], Alexander Brol[2], Andrea di Fonzo[1‡], Eddy Rijntjes[3], Josef Köhrle[3], Ulrich Schweizer[2], Clemens Steegborn[1*]

[1] Department of Biochemistry, University of Bayreuth, Universitätsstr. 30, 95447 Bayreuth, Germany
[2] Institut für Biochemie und Molekularbiologie, Universitätsklinikum Bonn, Rheinische Friedrich-Wilhelms-Universität Bonn, 53115 Bonn, Germany
[3] Charité-Universitätsmedizin Berlin, Corporate Member of Freie Universität Berlin and Humboldt-Universität zu Berlin, and Berlin Institute of Health, Institut für Experimentelle Endokrinologie, 10115 Berlin, Germany
[‡] Present address: Analytical Chemistry Facility, Istituto Italiano di Tecnologia, Via Morego 30, 16163 Genova, Italy
* Correspondence: clemens.steegborn@uni-bayreuth.de

Supplementary Figure S1

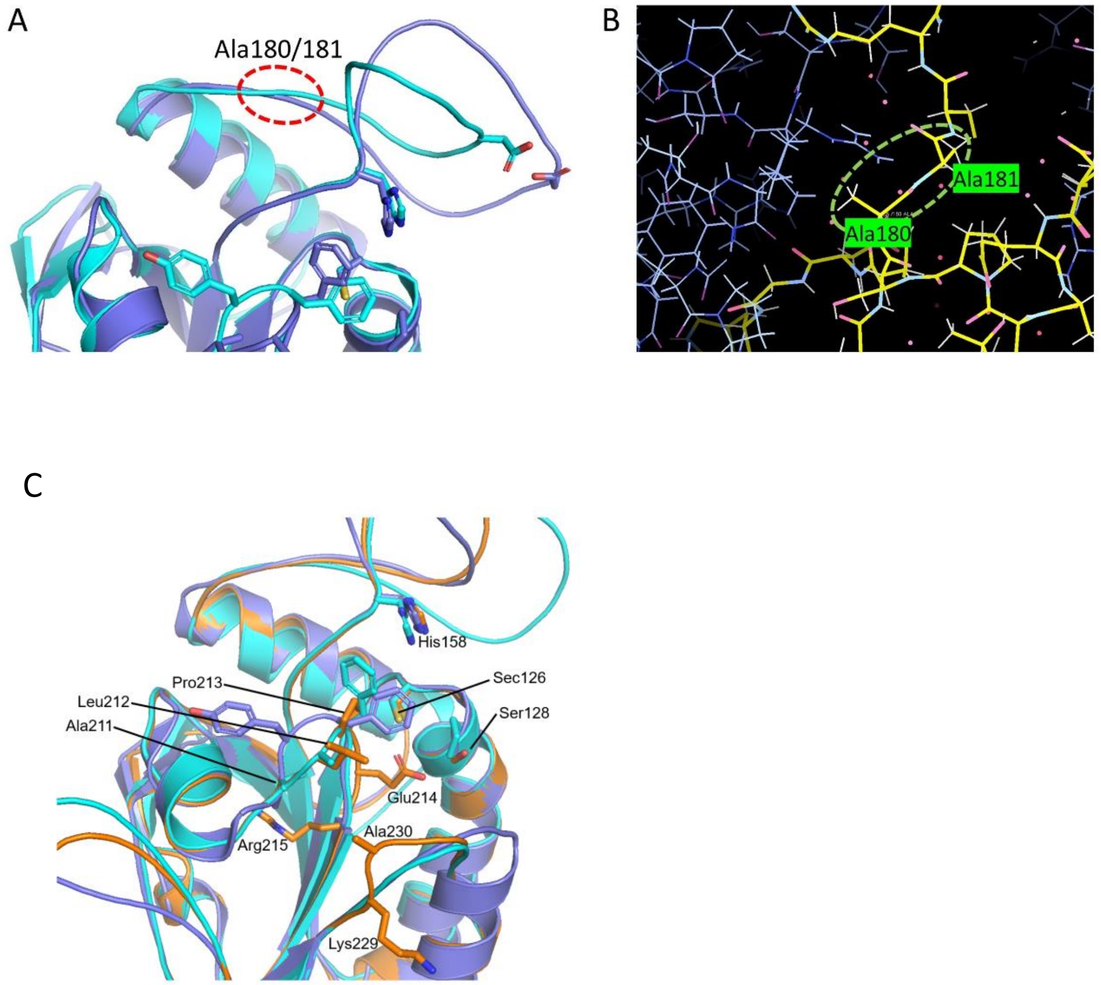

Figure S1: Dio2 LysLys->AlaAla mutation site and comparison of the active sites of mDio1, mDio2, and mDio3. (A) Overlay of the crystal structures of mDio2 (blue) and mDio3 (cyan), with the double mutation site LysLys->AlaAla in Dio2 labeled and indicated by the dotted line. **(B)** Crystal structure of mDio2 with the double mutation site LysLys->AlaAla labeled and indicated by the dotted line (yellow). Symmetry related mDio2 molecules are shown in blue. **(C)** Closeup of the active site regions of an overlay of the crystal structures of mDio2 (cyan) and mDio3 (blue) and the homology model for mDio1 (red).

Supplementary Figure S2

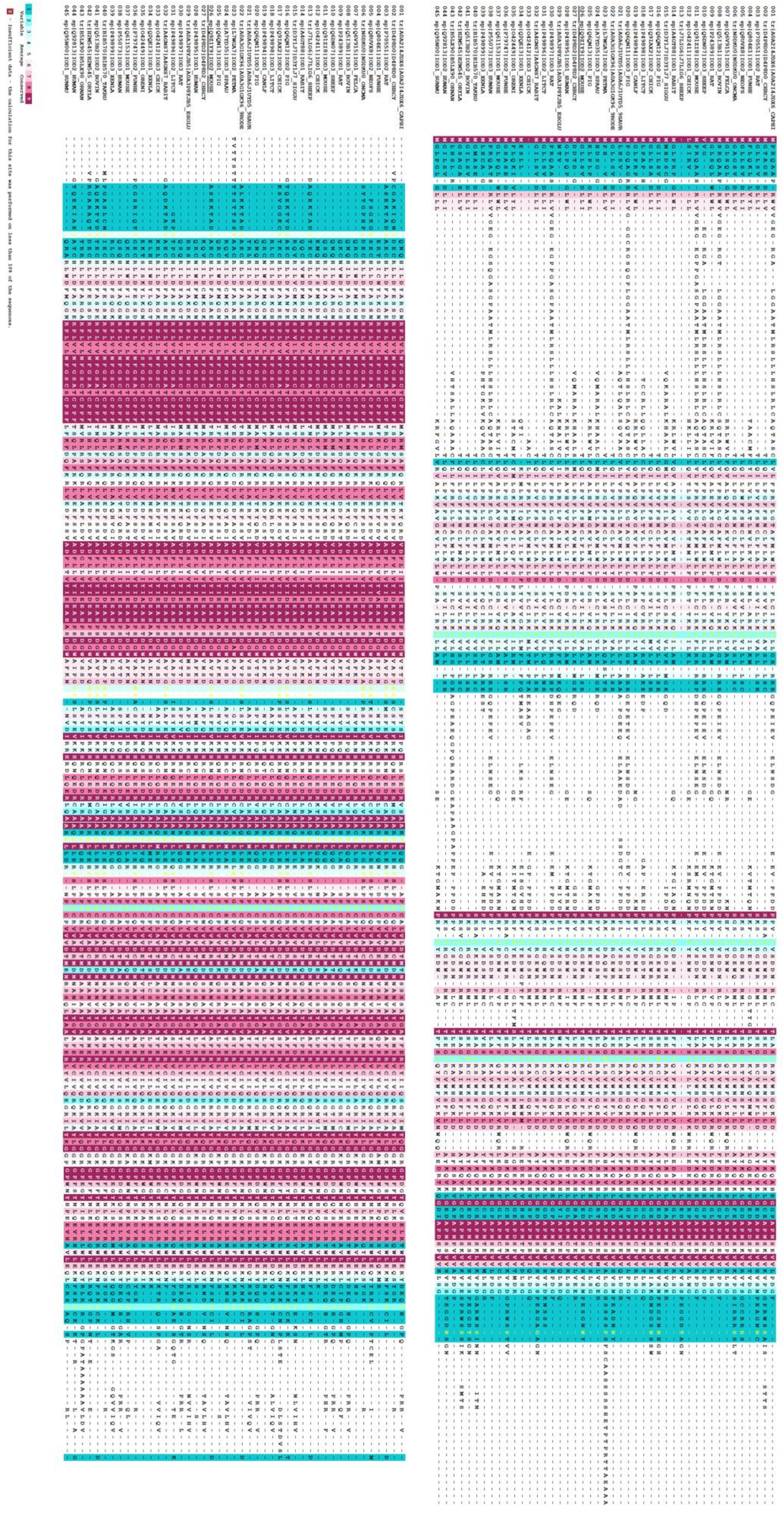

Figure S2: Alignment of Dio1-3 from several species.

Supplementary Figure S3

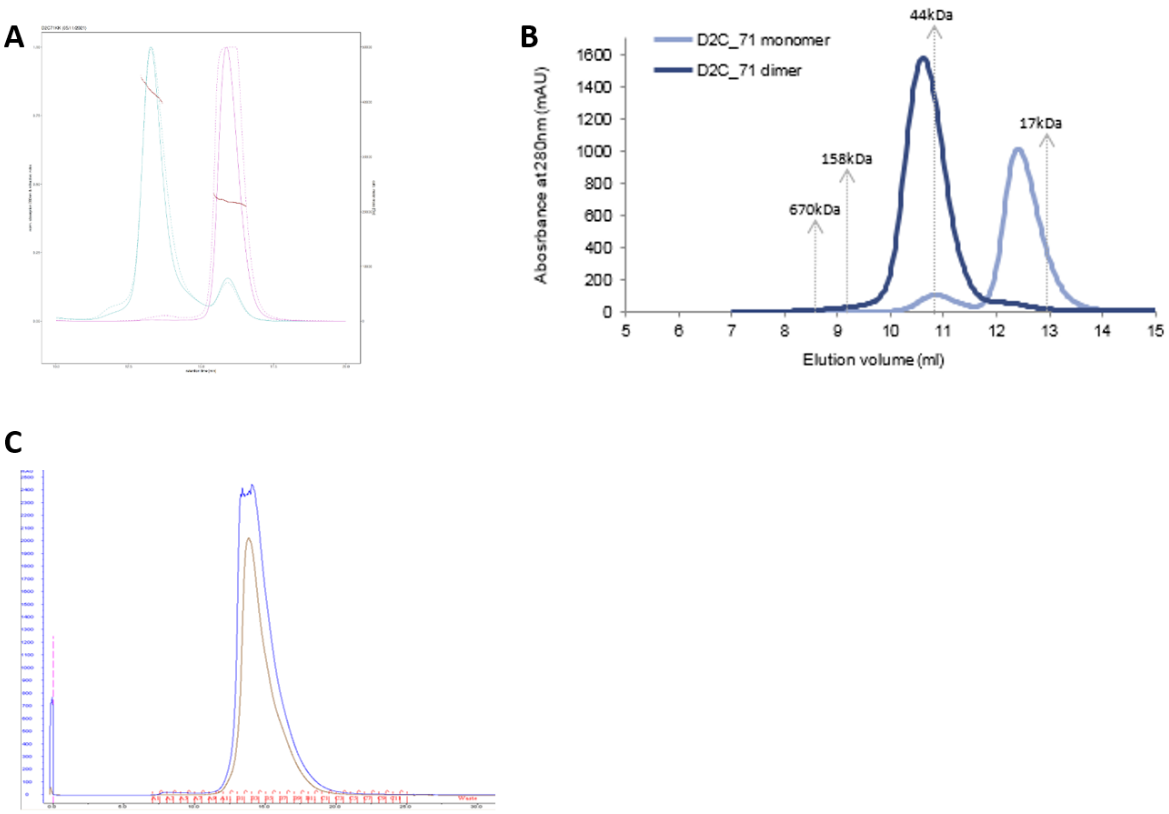

Figure S3: Size analyses for recombinantly produced mDio2 constructs. (A) MALS analysis indicates a monomeric and a dimeric species in preparations of mDio2-71-262. (B) SEC runs of isolated monomer or dimer of mDio2-71-262 did not show significant redistribution into these two species but resulted in single peaks corresponding to the injected species. (C) SEC for a longer construct mDio2-49-262 yielded a peak maximum corresponding to a dimer.

Supplementary Figure S4

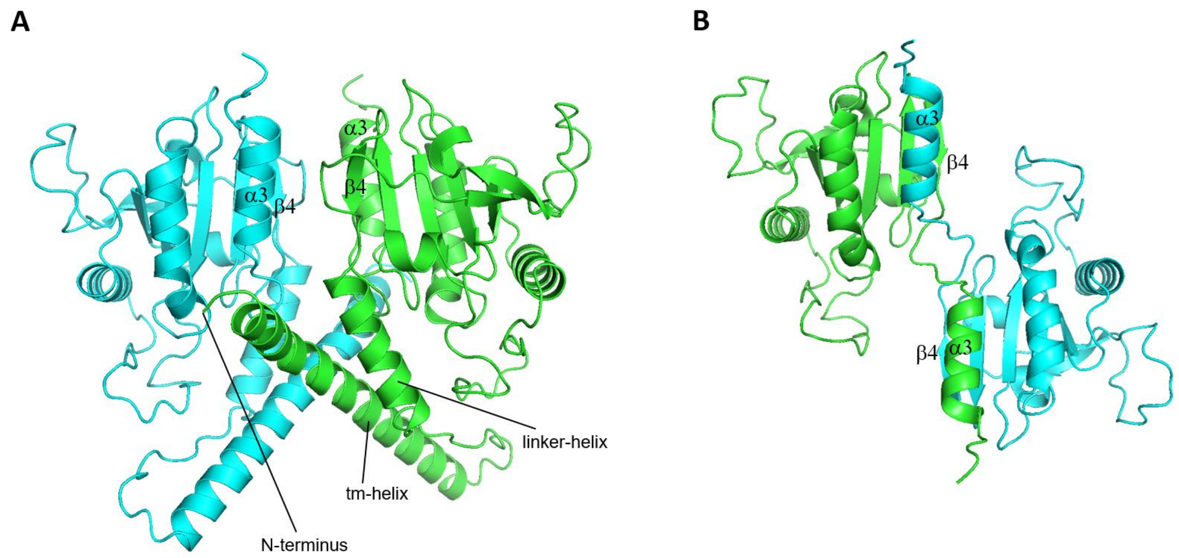

Figure S4: Modeling of mDio2 dimers. (A) Model for full-length mDio2 generated with a-Fold. (B) mDio2^{cat} dimer model with swapped C-terminal helices.

Supplementary Table S1

Table S1: PISA output on mDio2^{cat} crystal interactions

Interfaces XML View Details Download Search

##		Structure 1				×	Structure 2						interface	Δ^iG	Δ^iG	N_{HB}	N_{SB}	N_{DS}	CSS
NN	«»	Range	$^iN_{at}$	$^iN_{res}$	Surface Å^2		Range	Symmetry op-n	Sym.ID	$^iN_{at}$	$^iN_{res}$	Surface Å^2	area, Å^2	kcal/mol	P-value				
1	◉	A	119	29	9828	x	A	-y,x-y,z-1/3	2_554	92	23	9828	929.9	-2.9	0.578	1	5	0	0.000
2	○	A	81	24	9828	x	A	-y,x-y-1,z-1/3	2_544	81	23	9828	745.6	-1.5	0.596	3	3	0	0.000
3	○	A	20	6	9828	x	A	-y,x-y,z+2/3	2_555	13	2	9828	137.8	-1.8	0.240	0	0	0	0.000
4	○	A	14	6	9828	x	A	-y+1,x-y,z-1/3	2_654	14	5	9828	121.0	1.4	0.743	1	2	0	0.000
5	○	A	2	1	9828	x	A	x,y-1,z	1_545	1	1	9828	5.5	0.1	0.723	0	0	0	0.000

View Details Download Search
